# Increased vascular permeability is a surrogate marker of atherosclerotic plaque instability

**DOI:** 10.1186/1532-429X-17-S1-Q111

**Published:** 2015-02-03

**Authors:** Alkystis Phinikaridou, Marcelo E Andia, Begona Lavin Plaza, Prakash Saha, Alberto Smith, Rene Botnar

**Affiliations:** 1 Biomedical Egineering, King's College London, London, UK; 2 Radiology, Pontificia Universidad Católica de Chile, Santiago, Chile; 3 Academic Surgery, King's College London, London, UK

## Background

Hypercholesterolemia promotes endothelial dysfunction and neovascularization, which increase net vascular permeability and promote atherosclerosis. We have previously reported that gadofosveset, a clinically approved albumin-binding MR contrast agent, can be used to assess endothelial permeability, plaque progression/ regression in a murine model of atherosclerosis. Here, we used gadofosveset to investigate the effect of vascular permeability on plaque instability in a rabbit model of atherosclerosis.

## Methods

Atherosclerosis was induced in New Zealand White rabbits by cholesterol-diet and endothelial denudation. In vivo MRI of the abdominal aorta was performed at 1 and 12-weeks using a 3T Philips Achieva scanner and a 32-channel coil. Native and contrast enhanced images were acquired before and 40min after intravenous administration of 0.03 mmol/kg of gadofosveset (Lantheus Medical Imaging, USA). ECG-triggered 2D T1wBB images were acquired before contrast injection with: TR/TE=2heartbeats/5.4ms, low-high profile order, echo-train-length=6, BB-delay=350ms, FOV=120x85mm, matrix=384x270, resolution=0.31x0.31mm, slice thickness=4mm, slices=25 and averages=2. T1 mapping was performed 30 minutes after gadofosveset injection using a 3D modified Lock-Locker sequence with: TR/TE=3.5/1.8ms, FA =10°, FOV=58x45x80mm, matrix=116x97, reconstructed resolution=0.2x0.2mm, slice thickness=3mm, slices=15, averages=1. T1wBB images were used to calculate the plaque area by manually tracing the vessel wall contours using Osirix. T1 mapping images were used to calculate the R1 of the vessel wall on a pixel-by-pixel basis using in house software (Matlab).

## Results

**Figure 1:**
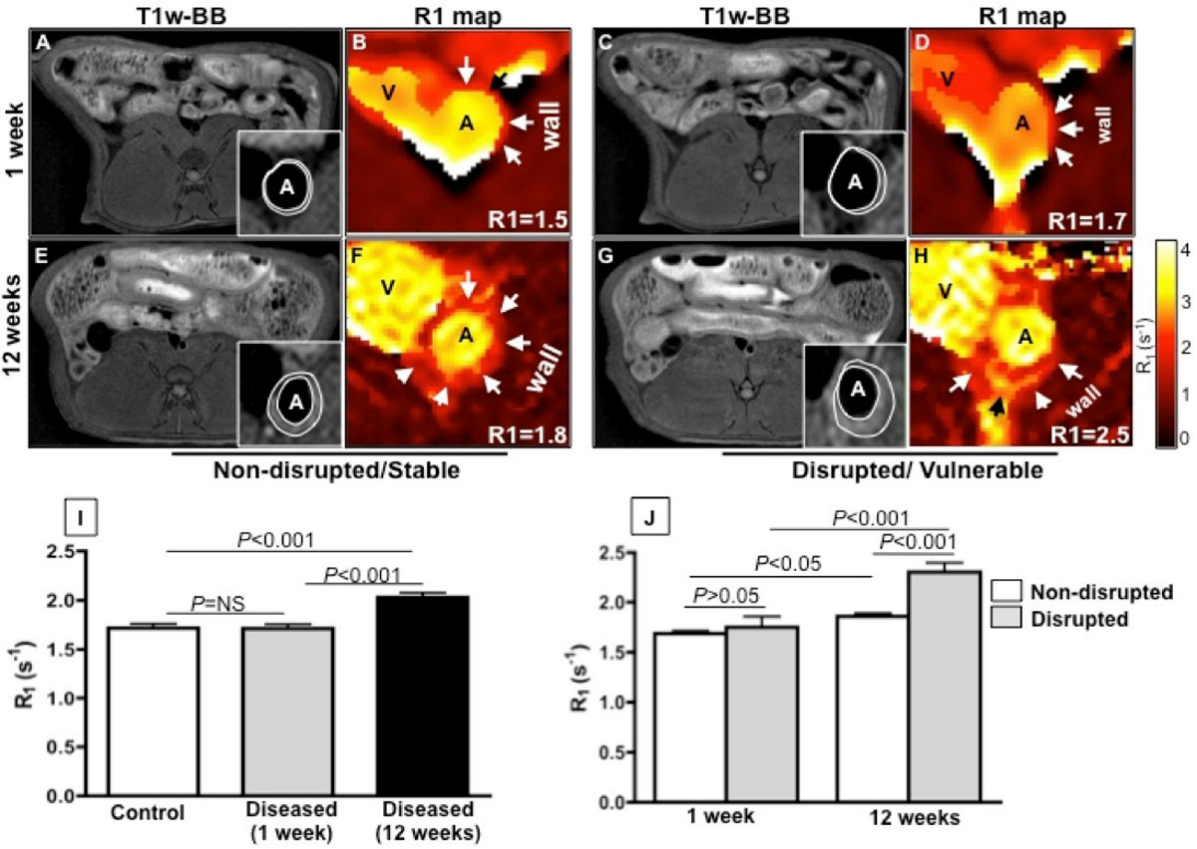
*In vivo* MRI of rabbit atherosclerosis at 3T. Vascular permeability increases with disease progression and is higher in vulnerable compared to stable lesions.

Native T1wBB images show increased vessel wall thickening due to plaque progression between 1 and 12 weeks, in both stable and vulnerable lesions (Fig. 1A, E and C, G). Corresponding R1 maps (Fig. 1B, D, F and H) show the aortic lumen as yellow because the contrast agent is still circulating in the blood stream and thus has a high R1. On the same R1 maps the vessel wall indicated by the arrows shows higher vessel wall relaxivity (orange color) with disease progression and particularly in vulnerable plaques at 12 weeks. Quantification of the vessel wall R_1_ showed significantly higher gadofosveset uptake with disease progression (Fig. 1I). Importantly, The increase in R_1_ was significantly higher in disrupted compared to non-disrupted lesions (Fig. 1J). Regression analysis showed that the R1 measured at 12weeks is an independent predictor of plaque instability (P=0.02, OR=6.96, CI = 1.3-37.2).

## Conclusions

Increased vascular permeability measured using an MR albumin-binding contrast agent and T1 mapping is a surrogate measure of plaque progression and instability and can be used to provide stratification of atherosclerotic disease.

## Funding

British Heart Foundation (PG/10/044/28343).

